# Rural–Urban Differences in Risk Factors for Motor Vehicle Fatalities

**DOI:** 10.1089/heq.2018.0006

**Published:** 2018-09-25

**Authors:** Carrie Henning-Smith, Katy B. Kozhimannil

**Affiliations:** Division of Health Policy and Management, Rural Health Research Center, University of Minnesota School of Public Health, Minneapolis, Minnesota.

**Keywords:** motor vehicle fatalities, rural, urban

## Abstract

**Purpose:** To examine rural–urban differences in motor vehicle fatality (MVF) risk factors.

**Methods:** We used 2017 County Health Rankings data to run stratified regression models to estimate county-level correlates of motor vehicle fatalities (MVFs) by rural and urban location.

**Results:** Rural counties have higher rates of MVFs than urban counties (22 vs. 14 per 100,000, *p*<0.001). Physical inactivity and uninsurance were associated with higher rates of MVFs, as was having a more racially or ethnically concentrated population and larger percentages of younger or older adults.

**Conclusion:** Interventions to reduce MVFs should take geographic location and population composition into account.

## Introduction

Motor vehicle fatalities (MVFs), combined with other types of unintentional injuries, are one of the five leading causes of death in the United States, and the leading cause of death among people of ages 1–44 years.^1,2^ Mortality rates from all five leading causes of death, including MVFs, are higher in rural areas than in urban areas.^1,2^ Furthermore, although the overall rate of MVFs is declining, it remains higher in rural areas than in urban areas.^3^ In addition to geography, MVF rates are also associated with sociodemographic characteristics, including race, ethnicity, age, and gender, which differ between rural and urban communities.^2,4–6^ For example, MVF rates are highest among American Indian/Alaskan Native populations, compared with all other racial and ethnic groups,^2^ men are more likely to die from motor vehicle crashes than women,^7^ and individuals under the age of 20 years and over the age of 69 years are more likely than those in middle age to have MVFs.^7^ Above and beyond individual characteristics, state policy context also matters for MVF rates; for example, states with stricter seat belt and texting-while-driving laws have lower rates of MVF.^6,8^ Less is known about whether rural–urban differences in MVFs persist after adjusting for population characteristics and state context and whether sociodemographic risk factors for MVFs differ by rural–urban location. This study addresses that gap and its results will be useful in tailoring interventions by geographic location to reduce MVFs risk.

## Methods

Data for this study come from the 2017 County Health Rankings, a compilation of county-level data produced by the University of Wisconsin with funding from the Robert Wood Johnson Foundation. We included all 2,709 counties for which there were complete data on the MVFs rate (86% of counties) and compared the MVFs rate by rural–urban location. We defined rural and urban location using Federal Office of Management and Budget definitions, categorizing all micropolitan and noncore counties as “rural” and all metropolitan counties as “urban.” We used ordinary least squares regression to model the association between rural–urban location and the MVFs rate, adjusting for various county-level sociodemographic characteristics. We stratified the analysis, running regression models separately for rural and urban location, to see whether risk factors for MVFs differed by geographic location. In all models, we included a fixed effect for state, as differences in state policies are associated with differences in MVF rates.^6,8,9^

## Results

For every 100,000 people, rural counties experienced eight more deaths by MVFs than urban counties per year (22 vs. 14, *p*<0.001); Table 1. Based on current population, this difference amounts to nearly 4000 additional lives lost per year in rural counties.

**Table 1.** **Correlates of Motor Vehicle Fatalities by Rural–Urban Location**

	*Full sample*			*Urban only*			*Rural only*		
*Observations (counties),* n	*2709*			*1124*			*1585*		
*Motor vehicle fatality rate (per 100,000)*	*18.80*			*14.03*			*22.18*		
	*Coefficient*	*SE*	p *value*	*Coefficient*	*SE*	p *value*	*Coefficient*	*SE*	p *value*
Rural (ref: Urban)	2.86	0.32	0.000						
Health behaviors									
Adult smoking	12.42	8.17	0.129	−9.90	8.31	0.233	21.17	12.44	0.089
Excessive drinking	−28.47	6.57	0.000	−15.17	6.08	0.013	−37.66	10.86	0.001
Insufficient sleep	−35.89	5.98	0.000	−40.99	5.59	0.000	−29.11	9.88	0.003
Physical inactivity	43.36	4.59	0.000	40.60	4.91	0.000	41.97	6.89	0.000
Adult obesity	1.97	4.89	0.687	10.97	5.05	0.030	−2.47	7.40	0.738
Socioeconomic characteristics									
Uninsured	31.28	4.39	0.000	29.67	4.71	0.000	31.84	6.49	0.000
Unemployment rate	−20.19	10.34	0.051	2.04	11.25	0.856	−27.59	15.54	0.076
Adults with some college	−6.52	2.20	0.003	−18.63	2.33	0.000	−0.07	3.36	0.983
Child poverty rate	2.42	3.49	0.489	−5.13	3.81	0.179	4.89	5.15	0.343
Food insecurity	4.31	7.05	0.541	10.80	6.76	0.110	3.30	11.41	0.773
Demographic composition									
Percentage under 18	55.41	7.20	0.000	10.97	7.10	0.122	81.13	11.26	0.000
Percentage 65 and older	42.65	6.23	0.000	12.22	6.09	0.045	58.19	9.81	0.000
Percentage black	38.46	10.29	0.000	11.79	10.65	0.268	43.96	17.95	0.014
Percentage Hispanic	28.61	10.11	0.005	−0.82	10.39	0.937	35.43	17.55	0.044
Percentage American Indian/Alaskan Native	61.97	10.73	0.000	35.62	11.88	0.003	63.84	18.61	0.001
Percentage Asian	23.54	14.45	0.103	−1.94	13.69	0.887	11.06	36.56	0.762
Percentage white	33.00	10.42	0.002	8.85	10.77	0.411	37.48	18.15	0.039
Percentage female	−49.53	8.28	0.000	−34.15	9.33	0.000	−63.18	12.37	0.000
Adjusted r^2^	0.51			0.60			0.35		

Source: Authors' analysis of 2017 County Health Rankings.

After adjusting for county-level characteristics, including health behaviors, socioeconomic characteristics, and demographic composition, rurality continued to be associated with a higher rate of MVFs, amounting to three additional deaths per 100,000 (*p*<0.001); Table 1. Higher rates of physical inactivity and uninsurance were associated with higher county-level rates of MVFs, as was having a larger percentage of the population <18 years old or >64 years old. Having a more racially or ethnically concentrated population was associated with higher rates of MVFs, with rates being especially high for counties with a higher percentage of American Indian/Alaskan Native individuals (b=61.97, *p*<0.001). Having more adults with some college and a greater proportion of females in the county was both associated with lower rates of MVFs. Contrary to our hypotheses, having higher rates of both excessive drinking and insufficient sleep was both associated with lower rates of MVFs in U.S. counties.

In stratified analyses by geography, many, but not all, of the risk factors for increased rates of MVFs remained consistent. Exceptions included adult obesity, which was associated with higher rates of MVFs in urban (*p*<0.05) but not rural counties. The percentage of adults with some college had a significant negative relationship with MVFs for urban (*p*<0.001) but not rural counties (*p*=0.983). Meanwhile, having a higher percentage of younger adults was associated with higher rates of MVFs in rural counties (*p*<0.001) but not urban (*p*=0.122). In general, the association between age, gender, racial, and ethnic composition and MVF rates was consistently larger and more significant in rural counties. The adjusted r^2^ for the three models indicated that more of the variance in MVF rates was explained in the full model (adjusted r^2^=0.51) and urban subgroup model (adjusted r^2^=0.60), compared with the rural subgroup model (adjusted r^2^=0.35), suggesting that other unobserved factors play more significant roles in predicting MVF rates in rural counties.

## Discussion

We found higher rates of MVFs among rural counties, confirming other research on this topic.^1^ We also found that those differences cannot be entirely explained by differences in health behaviors or sociodemographic composition of the population. However, our analysis was limited by available data, and there are likely several unobserved factors that contribute to differences in MVFs by geography. For instance, rural drivers tend to drive at higher speeds, are less likely to use seat belts, and are more likely to have older vehicles with fewer safety features, such as airbags.^10,11^ Furthermore, rural and urban areas differ in their perceptions of transportation challenges and rural residents are less likely than urban residents to limit their driving, even if they have a medical condition that makes it difficult to drive safely.^12^ This may be due to more limited availability of viable transportation alternatives, such as public transportation or affordable taxi services for rural residents.

Higher death rates in rural areas have caused alarm among public health practitioners and deserve urgent attention. MVFs are one of the leading causes of potentially preventable deaths in rural counties nationally and require broad policy support. Such policy interventions must go beyond state laws about seat belts, texting, and similar safety issues,^6,8,9^ all of which are important, but are unable to prevent all MVFs and to eliminate rural–urban disparities in MVF rates. Reducing MVF rates in rural counties will require a multifaceted approach, including addressing rural transportation infrastructure, access to healthcare, and emergency response capability.^2^

MVF rates also differ by population composition in rural and urban communities, and efforts to reduce MVFs should be tailored by both geographic location and population sociodemographics. Such efforts could include culturally and geographically tailored public health education campaigns and outreach, subsidized car seats and safety devices for low-income populations, additional options for public and active transportation, and programs designed to encourage safe driving—or alternatives to driving—for younger and older drivers. Ultimately, all fatalities due to motor vehicle accidents should be considered avoidable deaths and every effort should be made to prevent them, especially in rural areas, where the overall mortality burden is higher.^1^ Achieving health equity for rural residents will require addressing motor vehicle usage and fatalities as an important social determinant of health.

## Abbreviations Used

MVF: motor vehicle fatalities
